# A Lectin from *Dioclea violacea* Interacts with Midgut Surface of *Lutzomyia migonei*, Unlike Its Homologues, *Cratylia floribunda* Lectin and *Canavalia gladiata* Lectin

**DOI:** 10.1155/2014/239208

**Published:** 2014-11-05

**Authors:** Juliana Montezuma Barbosa Monteiro Tínel, Melina Fechine Costa Benevides, Mércia Sindeaux Frutuoso, Camila Farias Rocha, Francisco Vassiliepe Sousa Arruda, Mayron Alves Vasconcelos, Francisco Nascimento Pereira-Junior, João Batista Cajazeiras, Kyria Santiago do Nascimento, Jorge Luiz Martins, Edson Holanda Teixeira, Benildo Sousa Cavada, Ricardo Pires dos Santos, Margarida Maria Lima Pompeu

**Affiliations:** ^1^Laboratory of Parasitology, Faculty of Medicine, Federal University of Ceará, 60430-160 Fortaleza, CE, Brazil; ^2^Integrated Laboratory of Biomolecules (LIBS), Department of Pathology and Legal Medicine, Faculty of Medicine, Federal University of Ceará, 60430-160 Fortaleza, CE, Brazil; ^3^Laboratory of Biologically Actives Molecules, Biochemistry and Molecular Biology Department, Federal University of Ceará, 60440-970 Fortaleza, CE, Brazil; ^4^Institute of Chemical and Geosciences, Federal University of Pelotas Institute of Chemical and Geosciences, Federal University of Pelotas, 96160-000 Pelotas, RS, Brazil; ^5^Computer Engineering of Sobral, Federal University of Ceará, 62042-280 Sobral, CE, Brazil

## Abstract

Leishmaniasis is a vector-borne disease transmitted by phlebotomine sand fly. Susceptibility and refractoriness to *Leishmania* depend on the outcome of multiple interactions that take place within the sand fly gut. Promastigote attachment to sand fly midgut epithelium is essential to avoid being excreted together with the digested blood meal. Promastigote and gut sand fly surface glycans are important ligands in this attachment. The purpose of the present study was to evaluate the interaction of three lectins isolated from leguminous seeds (Diocleinae subtribe), D-glucose and D-mannose-binding, with glycans on *Lutzomyia migonei* midgut. To study this interaction the lectins were labeled with FITC and a fluorescence assay was performed. The results showed that only *Dioclea violacea* lectin (DVL) was able to interact with midgut glycans, unlike *Cratylia floribunda* lectin (CFL) and *Canavalia gladiata* lectin (CGL). Furthermore, when DVL was blocked with D-mannose the interaction was inhibited. Differences of spatial arrangement of residues and volume of carbohydrate recognition domain (CRD) may be the cause of the fine specificity of DVL for glycans in the surface on *Lu. migonei* midgut. The findings in this study showed the presence of glycans in the midgut with glucose/mannose residues in its composition and these residues may be important in interaction between *Lu. migonei* midgut and *Leishmania*.

## 1. Introduction

Cutaneous leishmaniasis is endemic in the tropics and neotropics. This pathology is often referred to as a group of diseases because of the varied spectrum of clinical manifestations, which range from small cutaneous nodules to gross mucosal tissue destruction [1]. Cutaneous leishmaniasis can be caused by several* Leishmania* spp. and is transmitted to human beings and animals by sand flies [2].

*Lutzomyia migonei* is one of the sand flies that transmit the American cutaneous leishmaniasis [3]. Since several sand flies are specific or even permissive to development of different* Leishmania* parasites,* Lu. migonei *can also be considered as a possible vector for American visceral leishmaniasis [4, 5]. According to Rangel and Lainson [6], this specie is widely distributed in South America, being found from Argentina to Colombia and living in a variety of habitats.

When the parasites are taken up with the blood meal, they undergo a period of replication and development in the midgut. Then, these parasites differentiate to an infective metacyclic stage, which is adapted for transmission to mammals [7]. The procyclic promastigotes are surrounded by the peritrophic matrix [8], which is constituted by a mixture of chitin, proteins, glycoproteins, and proteoglycans [9]. The next step for the vector infection is the insertion of parasites flagella into midgut microvilli [10]. The interaction between parasite and the vector midgut cells is controlled by species-specific modifications of the major surface glycoconjugate of promastigotes, lipophosphoglycan (LPG), which selectively binds to the midgut galectin receptor [7, 11, 12]. Nevertheless, previous works reported the presence of glycans in the midgut that serve as binding sites for lectins on* Leishmania* surface [13, 14].

Lectins are a group of widely distributed and structurally heterogeneous (glycol-) proteins that contain at least one noncatalytic domain, which selectively recognizes and reversibly binds to specific carbohydrates or glycoconjugates (glycoproteins and glycolipids) without altering the structure of the ligand [15].

Among plant lectins, those isolated from Diocleinae subtribe are well studied. These lectins have many chemical and physicochemical properties in common, such as the specificity for D-glucose/D-mannose residues [16]. Minor differences in the ratios of dimeric and tetrameric forms of the lectins, together with differences in the relative orientations of the carbohydrate-binding sites in the quaternary structures, have been hypothesized to contribute to the differences in biological activities exhibited by Diocleinae lectins [17]. These interesting properties make these lectins valuable biotechnological tools. Furthermore, Diocleinae lectins provide an excellent system to study the effects of minor changes in the protein structure on their functional properties in biological models [18].

Thus, in the present study three lectins isolated from the seeds of* Cratylia floribunda* (CFL),* Canavalia gladiata* (CGL), and* Dioclea violacea* (DVL) were labeled with fluorescein isothiocyanate (FITC) and evaluated concerning their ability to recognize glycans on* Lu. migonei* midgut.

## 2. Materials and Methods

### 2.1. Lectins Purification

Lectins from* Cratylia floribunda* (CFL),* Dioclea violacea* (DVL), and* Canavalia gladiata* (CGL) were purified as previously reported [19–21]. Briefly, the lectins were extracted from air-dried ground seeds collected in Fortaleza-CE, Brazil, and defatted with n-hexane. The protein extract was obtained by continuous stirring with 0.9% NaCl (1 : 10 w/v) at 20°C for 4 h, followed by centrifugation at 10.000 g for 20 min at 4°C. The supernatant was submitted to affinity chromatography on a Sephadex G-50 column (5 × 25 mm), equilibrated with 0.9% NaCl containing 5 M CaCl_2_ and 5 M MnCl_2_. Then, the column was washed using the same buffer at a flow rate of 45 mL/h. The bound lectin was eluted with 0.1 M glycine, pH 2.6; dialyzed extensively against distilled water; and lyophilized. The purity of each lectin was monitored by SDS-PAGE, as described by Laemmli [22].

### 2.2. FITC-Labeled Lectins

FITC-labeled CFL, DVL, and CGL were prepared in inhibition buffer (0.1 M D-mannose in M carbonate-bicarbonate buffer, pH 9.0), conjugation buffer (0.1 M carbonate-bicarbonate buffer, pH 9.0), and washing buffer (phosphate-buffered saline: 0.01 M sodium phosphate buffer, 0.027 M KCl, and 0.15 M NaCl, pH 7.4). Initially, the lectins were dissolved in inhibition buffer and incubated at 37°C for 1 h. Then, 250 *μ*L fluorescein isothiocyanate (FITC) (500 *μ*g/mL in conjugation buffer) was added dropwise. The solution was incubated for 2 h at room temperature under gentle stirring. Subsequently, unconjugated FITC was separated from FITC-lectin by size exclusion chromatography using a Sephadex G-25 column previously equilibrated and eluted with washing buffer. The absorbance of all fractions was determined at 280 nm (protein) and 495 nm (FITC) to verify the chromatographic efficiency. FITC-labeled lectins were then dialyzed against 0.1 M acetic acid for 1 h to remove the inhibitor carbohydrate and were extensively dialyzed against distilled water.

### 2.3. FITC-Labeled Lectins Blocked with D-Mannose

The same FITC-labeled lectins were blocked with D-mannose. Briefly, the FITC-lectins were incubated with 0.1 M D-mannose at 37°C for 30 min to block the CRD. The solutions were then filtered through a 0.22 *μ*m cellulose filter and stored for later use.

### 2.4. Sand Fly Capture, Culture Maintenance, and Midgut Dissection

The sand flies were collected in Baturité, a city located in the northern region of Ceará state, Brazil, which is endemic for cutaneous leishmaniasis. A colony of* Lu. migonei* was reared and maintained in the lab as described by Modi and Tesh [23]. Adult flies were maintained at 23°C and 95% relative humidity and had free access to a 10% sucrose solution on a cotton pad. Three-day-old females were fed once a week with a blood meal from an anaesthetized hamster. Experiments were performed with 3–10-day-old females that had not taken a blood meal. Sand flies (5-6 per group) were quickly immobilized at −20°C and individually dissected in drops of 0.1 M PBS, pH 7.4, under a stereoscopic microscope.

### 2.5. Midgut-Binding Assays

Midguts were transferred to cold PBS and fixed in a 4% paraformaldehyde/PBS solution at 4°C for 1 hour. The abdominal segments of the fixed midguts were opened using a fine needle. The midguts were then transferred to wells of fluorescence slides and washed 3 times with 5% BSA in PBS for 5 min. Then, they were separately incubated with blocked and unblocked FITC-lectins (100 *µ*g/mL) and maintained at 37°C for 40 min in a dark humid chamber. The negative control was performed with 5% BSA in PBS containing 0.5% of FITC. The midguts were then washed 3 times with PBS and transferred to fluorescence slides and observed in a fluorescence microscope.

### 2.6. Fluorescence Microscopy

Fluorescence images of midguts were obtained using a Nikon Eclipse 80i microscope, fitted with a 100 W mercury lamp, and a fluorescein dichroic cube filter (excitation 490 nm and emission 520 nm). Each image was taken using an objective of ×4 and ocular of ×10 (magnification of ×40). An Evolution MP 5.0 cooled camera (Media Cybernetics, Silver Spring, MD, USA) was used to acquire the images at 7.5 s of exposition. The negative control was used for the subtraction of effects of natural fluorescence of midguts and residual fluorescence of FITC. The same images were acquired in bright field for comparison with fluorescence images. The images were saved in tagged image file format (TIFF).

### 2.7. Fluorescence Intensity

Ten midguts treated with each lectin (blocked and unblocked) were evaluated to determine the mean fluorescence intensity. Fluorescence intensity was quantified through the arithmetic mean of the distribution of gray values within a given midgut image area, which was determined according to the following formula:

(1) $$\begin{array}{c} \text{Fluorescence intenisty}=\frac{\sum_{x}\sum_{y}\text{gray}\left(x,y\right)}{n}, \end{array}$$

where gray(*x*, *y*) is the gray value (0–255) of the pixel of *xy* coordinates and *n* is the number of pixels within a given midgut area. With TIFF images, the gray value is calculated by converting each pixel to gray scale using the formula [24]:

(2) grayx,y=0.299 redx,y+0.587 greenx,y+0.114 bluex,y,

where red (*x*, *y*), green (*x*, *y*), and blue (*x*, *y*) are red, green, and blue values (0–255) of the pixel of *xy* coordinates. ImageJ 1.32J software was used for fluorescence intensity calculation [25].

### 2.8. Statistical Analysis

Statistical data analysis was based on the descriptive statistic-mean value; standard deviation (SD); margin of error (*E*); the Shapiro-Wilk test, to determine whether or not a random sample of values follows a normal distribution; and hypothesis test, to determine the significance of differences between groups. The margin of error (*E*) is the maximum likely difference between the observed sample mean and the true value of the population mean. When the population mean is unknown, *E* is determined according to following formula:

(3) $$\begin{array}{c} E=t_{\alpha /2}\frac{\text{SD}}{\sqrt{n}}, \end{array}$$

where *α* is the significance level, *n* is the number of samples, and *t*_*α*/2_ is Student's *t*-distribution parameter. The significance level used was 0.05. The software Statistica 10 (Statsoft Inc. 2011) was used in all statistical analysis.

## 3. Results and Discussion

In the present study three different lectins (CFL, CGL, and DVL), isolated from seeds of leguminous, were stained with FITC and evaluated regarding their ability to interact with glycans disposed on the surface of midgut cells of* Lu. migonei*.

**Figure 1:**
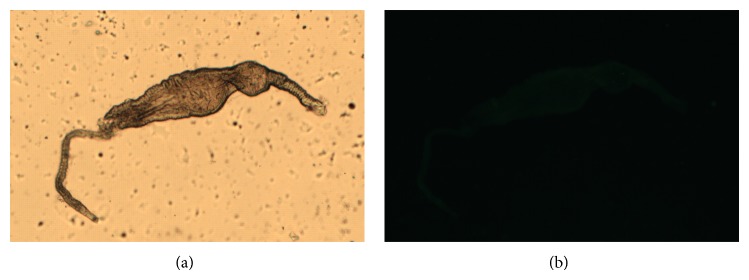
Bright field (a) and fluorescence (b) images of the control sample.

**Figure 2:**
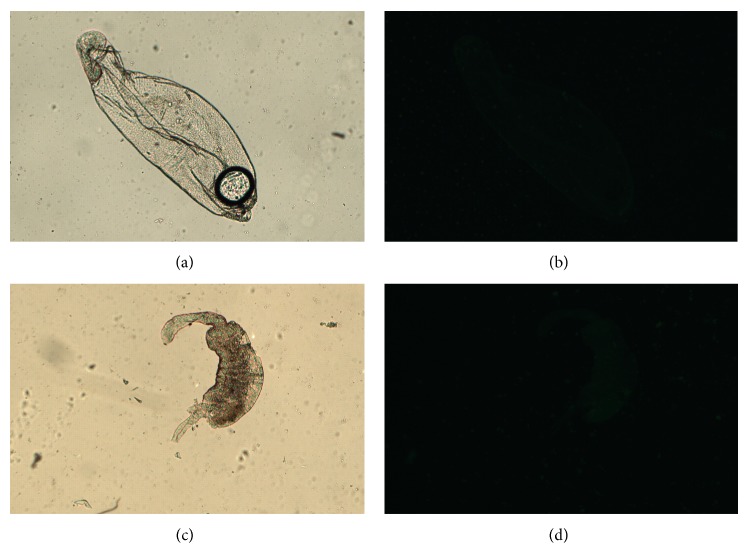
Bright field (a) and fluorescence (b) images of the FITC-CFL. The same images were acquired with the FITC-CFL blocked with D-mannose (c) and (d).

**Figure 3:**
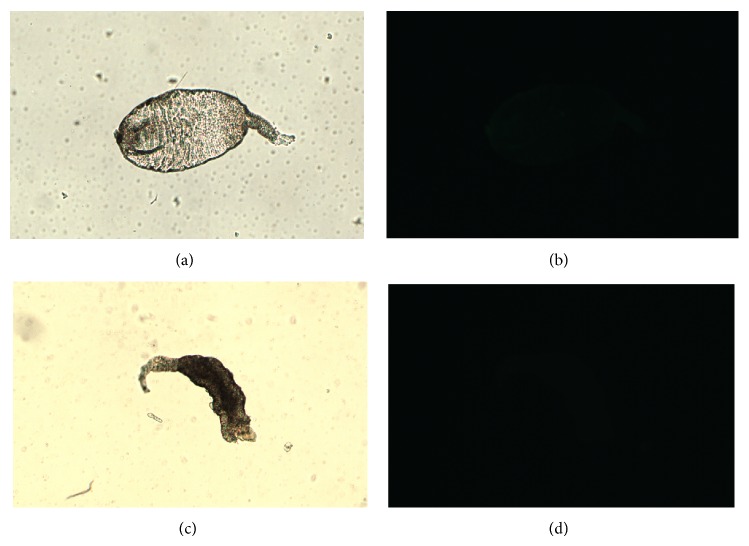
Bright field (a) and fluorescence (b) images of the FITC-CGL. The same images were acquired with the FITC-CGL blocked with D-mannose (c) and (d).

**Figure 4:**
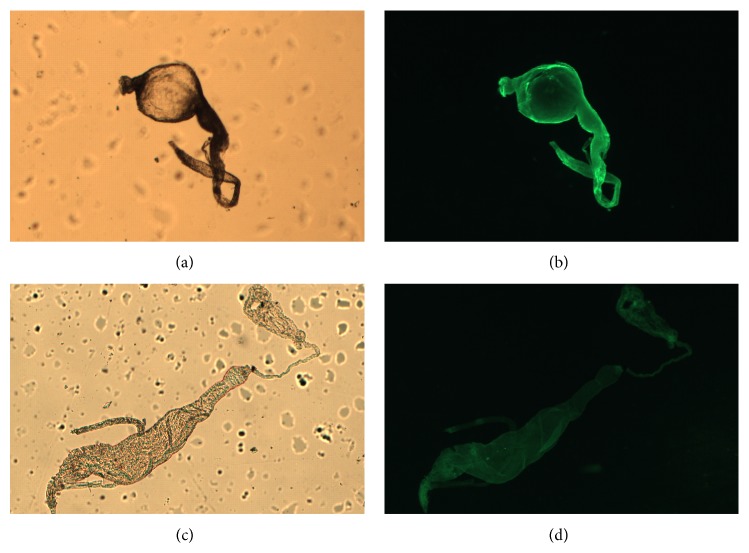
Bright field (a) and fluorescence (b) images of the FITC-DVL. The same images were acquired with the FITC-DVL blocked with D-mannose (c) and (d).

The midguts were disposed in fluorescent slides and observed in bright field microscopy for the comparison with fluorescence images. The bright field images are showed in Figures 1(a), 2(a), 2(c), 3(a), 3(c), 4(a), and 4(c). Regarding the control samples (midguts without treatment with lectins), the images showed weak intrinsic fluorescence (Figure 1(b)).

Similar fluorescence intensity was achieved when the midguts were treated with CFL and CGL without D-mannose (Figures 2(b) and 3(b), resp.). Interestingly, unlike CFL and CGL, in the treatment with DVL a strong fluorescence was observed (Figure 4(b)). In order to verify if the lectins interact with midgut cells through the CRD, all lectins were blocked with D-mannose and new images were acquired. No differences were observed in the fluorescence pattern of blocked and unblocked CFL and CGL (Figures 2(d) and 3(d), resp.). On the other hand, when the midguts were treated with blocked DVL, a remarkable decrease in the fluorescence pattern was achieved (Figure 4(d)).

**Figure 5:**
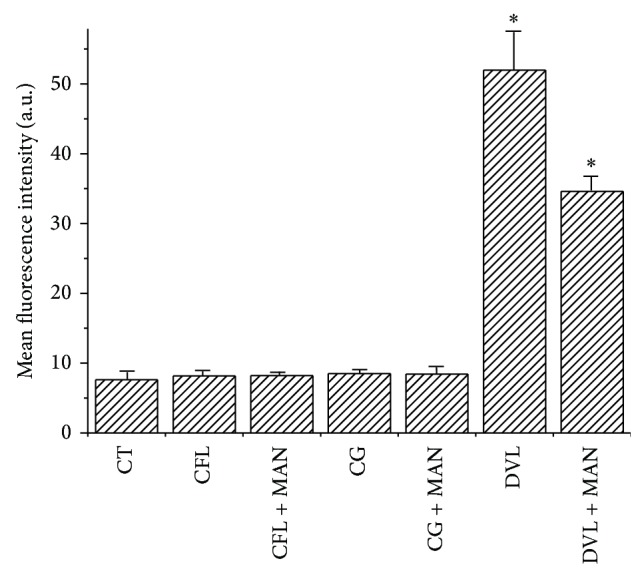
Fluorescence intensity of blocked and unblocked FITC-lectins. The data are expressed as means ± error. ^*^ *P* < 0.05 related to the control and ^#^ *P* < 0.05 related to FITC-DVL.

Moreover, data from mean fluorescence intensity of midguts were collected for control, blocked, and unblocked lectins. The mean fluorescence intensity (arbitrary units (AU)) of the control was 7.62 ± 1.29. Concerning FITC-lectins, CFL, CGL and DVL, showed, respectively, 8.17 ± 0.94, 8.50 ± 0.69, and 51.95 ± 5.76. The fluorescence intensities exhibited by the same lectins blocked with D-mannose were 8.20 ± 0.85, 8.43 ± 1.35, and 34.618 ± 2.42 (Figure 5).

A statistical analysis using Shapiro-Wilk test resulted in normal distributions for all experimental groups. Moreover, the ANOVA test (with Tukey* post hoc*) was used to verify significant statistical differences among the means. In this analysis, it was observed that blocked and unblocked DVL showed significant statistical differences when compared to the control. In addition, statistically significant differences were achieved among themselves (Figure 5).

Plant lectins have been used as biotechnological tools in different areas. These areas include cancer detection [26], effects on bacterial and yeasts growth as well as their biofilms [27, 28], anti-inflammatory and antinociceptive effect [29], anti-HIV action [30], antidepressant-like effect on nervous cells [31], larvicidal activity of* Aedes aegypti* [32], antineoplastic agents [33], prohealing effect [34], immunization against* Leishmania* infection [35], carbohydrate identification on* Leishmania* promastigotes [36], among others.

Since the interaction between lectins and carbohydrates is very specific, they can be used as tools to identify carbohydrates on the cell surface and thus decipher the glycocode of cell receptors. Therefore, in the present work three different lectins were assayed concerning their ability to identify glucose/mannose motifs on glycans from the epithelial surface of* Lu. migonei* midgut.

The recognition of host epithelia surfaces is an important step for infection by pathogens [37]. In many cases, proteins such as lectins are the mediators of adhesion events [7, 11, 12]. Despite the importance in elucidating the glycans that participate with the interaction between pathogen and vector midgut epitelia, only few studies describe the vector glycans that are involved in the interaction with adhesins of pathogens. On the other hand, many studies are focused on the role of LPG as the major adhesin responsible for midgut binding in all* Leishmania* species [38, 39]. Myskova and colleagues [14] using* Helix pomatia* lectin and Concanavalin A to detect glycoproteins in midgut lysates suggested a new modality of interaction between vector midgut and* Leishmania* pathogen.

According to the present study, only DVL interacted with glycans (glycoproteins or glycolipids) disposed on the midgut epithelial cell surface, suggesting the presence of glucose/mannose residues. Probably, glucose/mannose residues are important for the occurrence of the interaction between the vector and pathogen.

The lectins used in this study were isolated from members of Diocleinae subtribe and share a high degree of structural similarity between them [16]. However, only DVL was able to interact with midgut glycans of* Lu. migonei*, suggesting that minor changes in the structure of CRD are responsible for the basis of interaction between lectin and ligand. These changes include differences in the distance exhibited by specific amino acid residues that compose the primary CRD or/and alterations on the volume of this site [18].

## 4. Conclusions

In summary, both CFL and CGL were not able to interact with* Lu. migonei* midgut glycans. On the other hand, DVL established interactions with glycans as evidenced by fluorescence images. The interaction was inhibited when the lectin was blocked with D-mannose, suggesting the presence of terminal mannose residues on high mannose-, hybrids-, and complex-type of N-linked glycans on* Lu. migonei* midgut surface. Therefore, the present study corroborates with the literature about the composition of glycans involved in the interaction between* Leishmania* parasites and vectors. Future studies should propose the role of DVL as a biotechnological tool to inhibit specific interactions between* Leishmania *and digestive tract of the sand fly.
